# Resistance to TST/IGRA conversion in Uganda: Heritability and Genome-Wide Association Study

**DOI:** 10.1016/j.ebiom.2021.103727

**Published:** 2021-12-04

**Authors:** Michael L McHenry, Penelope Benchek, LaShaunda Malone, Mary Nsereko, Harriet Mayanja-Kizza, W. Henry Boom, Scott M. Williams, Thomas R. Hawn, Catherine M. Stein

**Affiliations:** a Department of Population & Quantitative Health Sciences, Case Western Reserve University, Cleveland, OH, USA; b Department of Medicine, Case Western Reserve University, Cleveland, OH, USA; c Department of Medicine, School of Medicine, Makerere University, Kampala, Uganda; d Department of Medicine, University of Washington, Seattle, WA, USA

**Keywords:** resistance to tuberculosis infection, M. tuberculosis, GWAS, phenotype definition, TB outcomes

## Abstract

**Background:**

Pulmonary tuberculosis (TB) is one of the most deadly pathogens on earth. However, the majority of people have resistance to active disease. Further, some individuals, termed resisters (RSTRs), do not develop traditional latent tuberculosis (LTBI). The RSTR phenotype is important for understanding pathogenesis and preventing TB. The host genetic underpinnings of RSTR are largely understudied.

**Methods:**

In a cohort of 908 Ugandan subjects with genome-wide data on single nucleotide polymorphisms, we assessed the heritability of the RSTR phenotype and other TB phenotypes using restricted maximum likelihood estimation (REML). We then used a subset of 263 RSTR and LTBI subjects with high quality phenotyping and long-term follow-up to identify DNA variants genome-wide associated with the RSTR phenotype relative to LTBI subjects in a case-control GWAS design and annotated and enriched these variants to better understand their role in TB pathogenesis.

**Results:**

The heritability of the TB outcomes was very high, at 55% for TB vs. LTBI and 50.4% for RSTR vs. LTBI among HIV- subjects, controlling for age and sex. We identified 27 loci associated with the RSTR phenotype (P<5e-05) and our annotation and enrichment analyses suggest an important regulatory role for many of them.

**Interpretation:**

The heritability results show that the genetic contribution to variation in TB outcomes is very high and our GWAS results highlight variants that may play an important role in resistance to infection as well as TB pathogenesis as a whole.

**Research in context**

**Evidence before this study**

Pulmonary tuberculosis (TB) is an infectious respiratory disease caused by the bacterium *Mycobacterium tuberculosis*, one of the most deadly pathogens on earth. TB primarily affects people living in developing countries, and has a particularly high burden in Southeast Africa. Prior studies suggest that most people have resistance to developing symptoms and most of those infected do not show apparent signs of infection (despite harbouring the bacteria). However, prior research suggests that some individuals are resistant to becoming infected at all, despite consistent exposure to the bacteria, and these people have been termed resisters (RSTR). Prior evidence suggests that genetic variation in the human hosts likely plays a role in this resistance to infection but the extent to which this resistance is genetically determined, and which variants are most important is in need of greater study.

**Added value of this study**

Proper study design and follow-up is very important to characterizing RSTR individuals and prior studies have not always been performed with adequate follow-up and measurement and misclassification is of concern in RSTR studies. This study has used the longest follow-up and the most accurate way of determining resistance to quantify the genetic contribution to resistance and identify the DNA variants that may play a role in conferring it. Understanding why some individuals are resistant may inform efforts to prevent TB through vaccines and treat TB through more effective therapies.

**Implications of all the available evidence**

We have quantified the genetic contribution to (i.e., the heritability of) resistance to infection, in addition to the heritability of other important TB outcomes. We have also identified specific DNA variants which may play an important role in the biological processes that govern resistance, helping to improve our understanding of how to prevent and combat TB infection and thus reduce the large global burden it imposes.

Alt-text: Unlabelled box

## 1 Introduction

Pulmonary tuberculosis (TB) is a major public health problem, as it causes more deaths than any other single infectious agent prior to the COVID-19 pandemic [1]. It is also the leading cause of death among people infected with human immunodeficiency virus (HIV) [2]. The bacterium, *Mycobacterium tuberculosis* (MTB) that causes most TB is transmitted via airborne droplets from coughing and sneezing by people with active disease. However, most people exposed to MTB do not develop active disease. In 2017, only 10 million people developed active disease and 1.6 million people died despite there being ∼1.7 billion latently infected people in 2014 [1,3]. These numbers are of interest because they demonstrate that the vast majority of people have resistance to active disease.

In addition to resisting active disease, some individuals do not develop traditional LTBI with a positive TST or IGRA immunologic response, even in the face of prolonged and persistent exposure to an infectious TB case [4,5]. These individuals, who may resist or clear MTB infection, or acquire infection with a non-IFNγ−centric T-cell response, have been termed resisters (RSTRs) [6,7]. Estimates of prevalence of RSTR varies as a function of follow-up time and method of diagnostics (i.e., tuberculin skin test (TST) and/or interferon gamma release assay (IGRA)), with current estimates in high-exposure settings ranging from 7-25% [6,[8], [9], [10], [11], [12]]. This phenotype is important for understanding pathogenesis and is relevant in designing potential vaccines and prevention strategies against TB, as it can provide insight into how individuals respond to prevent infection by MTB, a critical first step to developing disease [7,13]. The present study can expand on our current understanding of TB heritability greatly by 1.) utilizing a more accurate classification of the RSTR phenotype and 2.) analysing a wider range of TB phenotypes than previous studies.

Several studies have shown that host genetic factors can play a role in susceptibility/resistance to TB disease, but few have studied resistance to infection [14]. There does appear to be a genetic component to resistance to infection as our previous study of persistently TST-negative household contacts estimated heritability of this phenotype at 21.7% [15]. Also, purified protein derivative (PPD) reactivity is correlated among siblings, but not among unrelated children who live in the same household with similar exposures to MTB [16]. Such data are indicative of a genetic component to the RSTR phenotype. In addition, linkage analyses, candidate gene studies, and genome-wide association studies (GWAS) have added to the evidence that genetic variation associates with MTB infection [14,15,[17], [18], [19], [20], [21], [22], [23], [24], [25]), though very few have examined RSTR based on long-term follow-up and using both TST and IGRA for phenotype designation. Genetic influences on resistance to infection have been studied in two ways [5,6,8,9]. Some studies have examined LTBI as the trait of interest, using cross-sectional study design, without any long-term follow-up. Other studies have conducted longitudinal follow-up to identify individuals who started as uninfected (TST or IGRA negative), but eventually converted to TST/IGRA positive or LTBI. We have argued that RSTRs cannot be defined based on a single assessment without long-term follow-up, as conversion to test positivity can occur later, and the clinical significance of individuals that are discordant on the TST and IGRA is not well understood [10].

Previous studies have calculated the heritability for TST positivity but the heritability estimates for other possible outcomes in the pathogenesis of TB have not been established. There are several different possible outcomes once an individual has been exposed to MTB. Post exposure to MTB, some people exhibit signs of infection, while others never become infected [13]. In a small number of people, there may be early clearance of the bacteria [8,26,27]. If the infection is controlled, the host enters a stage of latent (i.e. asymptomatic) infection. This contrast between resistance to/clearance of the infection is the focus of our association analysis. In about 5-10% of those latently infected, the host progresses to active TB [28,29]. The extent to which genetic variation affects this transition can be examined by comparing active TB cases to LTBI subjects with a heritability estimate. These two transitions post-exposure (clearance/resistance relative to infection and active infection relative to latent) can be understood by different contrasts in heritability estimates to determine the extent to which each is genetically influenced. Additionally, there is a third contrast of interest that is not representative of a single step in TB pathogenesis but is in line with much of the prior literature surrounding genetic association studies of susceptibility to TB. Susceptibility is most often represented as a binary outcome where people with active TB are compared to those without. Thus, we can represent this contrast by comparing active TB cases to all others (including RSTRs, LTBI, and those who cannot be definitively classified as either).

The purposes of this study are: 1.) to establish the extent to which different TB phenotypes, including the RSTR phenotype, are influenced by genetic variation (i.e. the genetic heritability) and 2.) to identify individual variants that are associated with the RSTR phenotype relative to LTBI based on the results of a recently published long-term follow-up study that stringently characterized the LTBI and RSTR phenotypes.

## 2 Methods

### 2.1 Subject ascertainment and characterization of phenotypes

Subjects were ascertained as part of the Kawempe Community Health Study in Kampala, Uganda; a subset that was limited to RSTR and LTBI subjects were included in a long-term follow-up study, as previously described by Stein et al. [30,31]. Subjects were initially enrolled between 2002-2012, and the follow-up study was conducted from 2014-2017. All TB cases were culture-confirmed based on isolation of MTB from clinical gastric or sputum samples in the original study. Six subjects that were RSTR or LTBI in the original study but had developed symptoms prior to follow-up were not enrolled in this study. Household contacts of index TB cases were confirmed to have lived with the index case for at least 7 consecutive days during the previous 3 months in the original (2002-2012) study and were followed for at least 12 months as part of the original study. Latent MTB infection (LTBI) was determined based on a positive TST during the initial study and positive TST and IGRA during the follow-up study (and no symptoms of active TB). Resistance to infection (i.e. the RSTR phenotype) was defined as having consistently negative TST tests despite confirmed exposure in the original study and remaining TST/IGRA negative during the follow-up study, an average of 9 years. Individuals who were TST negative or TST positive during the original study but not included in the follow-up study (which only included the RSTR and LTBI subjects with follow-up IGRA testing as well as a determination if the subject ever developed active TB) are referred to as “no active TB” in this analysis, because they did not have the IGRA results necessary to confirm their LTBI or RSTR status. This was done for two reasons. First, discordance between the TST and IGRA tests has previously been demonstrated [31]. This introduces the possibility that TST+ subjects might actually be IGRA-negative. Secondly, persistently TST negative individuals may have converted to LTBI since their initial negative TST and therefore could not be accurately classified as RSTRs. Thus, the LTBI and RSTR individuals in this study were all confirmed as such in both the original and follow-up studies mentioned above. This helps maintain a consistent and accurate definition of the RSTR phenotype in our study which minimizes misclassification [10].

**Figure 1:**
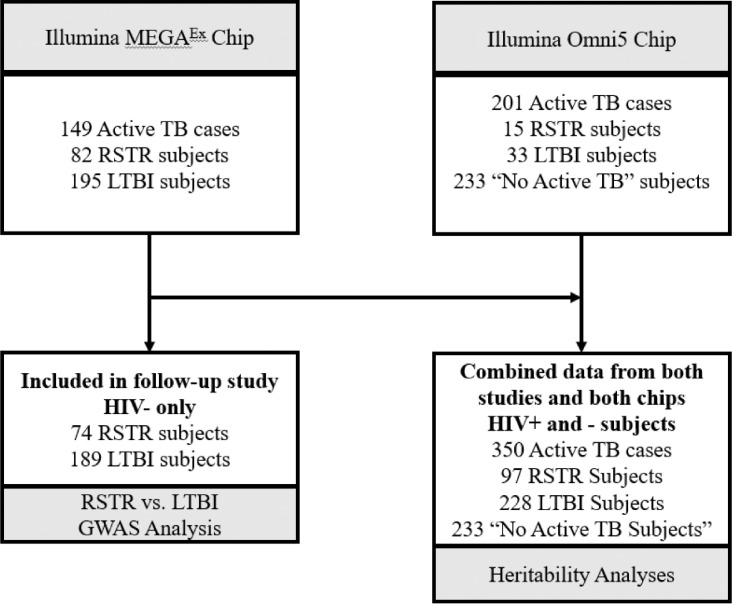
Source of Subjects and Genotype Data Diagram depicting the source of genotype data and number of subjects with each phenotype definition in the heritability and GWAS analyses.

In order to address our hypotheses, we used different but overlapping study samples from the same population in our two types of analyses. The first set of analyses aimed to determine the genetic contribution to various TB phenotypes (i.e. the heritability analyses), including but not limited to RSTR and LTBI. We chose four different phenotypic comparisons because they represent different biological processes in the pathogenesis of tuberculosis. Figure 1 shows the number of subjects with each phenotype definition in the heritability estimates, which included 97 RSTR, 228 LTBI, 350 Active TB, and 233 “No Active TB” subjects. Our first comparison, contrasting the 350 active TB subjects with all our other phenotypes, is similar to what has been called TB susceptibility in prior genetic studies, i.e. the probability of developing active TB relative to the rest of the population who do not have active TB. This is important to estimate as it allows the quantification of the genetic component of disease susceptibility that has been the major focus in TB genetics literature [13]. Our estimate is also unique in that we were able to confirm similar exposure for all subjects in the study.

Our second comparison, representing the heritability of being resistant to infection by MTB relative to those who are latently infected by MTB (but do not have active symptoms), allows us to quantify the genetic component for those individuals whose immune systems are not sensitized with positive TST/IGRA responses, the traditional markers of LTBI with an IFNγ dominant response. These individuals may prevent or clear infection entirely, or perhaps mount a IFN-γ independent response. This was done by contrasting the 97 RSTR subjects with the 228 LTBI subjects. The third contrast we made compared the 97 RSTR subjects with both the 228 LTBI subjects and the 350 active TB cases. This contrast groups the LTBI and active TB cases together as they have both been infected with MTB (and are thus not RSTR). Most if not all of the active TB cases are presumed to have gone through a stage of latent infection prior to having active TB and thus should, in theory, represent a group was not able to resist initial infection or clear the bacteria early.

Our fourth and final comparison, of active TB relative to LTBI, allows us to examine the genetic contribution to developing TB symptoms subsequent to infection relative to those who are infected but remain asymptomatic. This included our 350 active TB cases relative to the 228 LTBI subjects. Thus, the interpretation of each estimate sheds light on a potentially unique biological process and important step in the pathogenesis of TB. The one comparison that does not mirror one of the transitions in the pathogenesis of TB is the analysis of active TB cases vs. all other phenotypes. However, this analysis mirrors prior studies of TB susceptibility (which represent the majority of genetic association studies performed in TB), and allows us to produce a heritability estimates that used the same phenotype as that in prior literature so we can understand this heritability estimate in the context of prior literature.

Thus, these analyses included subjects who were enrolled in the follow-up study described above but also required additional subjects from the original KC Health study. This analysis included active TB cases and subjects who were not infected with active TB but could not be confirmed as RSTR based on the reasons outlined above. These analyses also included subjects who were genotyped with a different method (the Illumina Omni5 chip) than those included in the GWAS analysis, as this was necessary to obtain more subjects with active TB and those who were uninfected. The subjects from each chip included in the particular analysis are highlighted in Figure 1.

The second analysis was a GWAS that only included the 74 RSTR and 189 LTBI individuals who were genotyped on a single chip (the Illumina Infinium MEGA^EX^ chip) and included in the recently published long-term follow-up study; using long-term follow-up significantly reduced the chances of misclassification of the phenotype. This was done as we believe this is the most stringently defined phenotype of any GWAS study to date for RSTRs and as such may be best able to detect variants associated with the phenotype. This analysis examines the contrast between subjects who had a persistent asymptomatic infection (LTBI) and were concordantly TST+/IGRA+, and those who either resisted infection initially or were able to clear the infection entirely (RSTR) who were concordantly TST-/IGRA-. We also considered the possibility of operationalizing our outcome in the GWAS study as a case-control design where RSTR subjects are cases and both active TB subjects and LTBI subjects are considered controls (i.e. those who are not resistant to infection). However, we observed that the active TB sample includes subjects that are not concordant with respect to TST and IGRA. Thus, there is a greater possibility for misclassification which does not exist when we only examine the RSTR vs. LTBI with high quality follow-up data. Further, our heritability estimates revealed that the heritability of RSTR vs. LTBI is greater than that of RSTR vs. LTBI and active TB cases. We believe this is due to the higher potential for misclassification when the active TB cases are included.

### 2.2 Genotyping and QC

For the GWAS analysis, DNA samples were typed for the ∼2 million markers on the MEGA^EX^ chip. This chip was used for the RSTR and LTBI subjects included in the follow-up study as well as a set of active TB cases from the original study. Genotypes were called using Illumina's Genome Studio, version 2.0. Duplicates and indels were removed prior to QC. Data cleaning was done to remove samples with a call rate <0.97 and SNPs were removed if they had a call rate <0.95, a minor allele frequency < 0.05, or that deviated from Hardy Weinberg Equilibrium (*p* < 10^−6^ for deviation from HWE across all samples). After the QC thresholds described above and prior to analysis 733,040 markers remained.

Gender was estimated for all subjects by the Genome Studio Software. Subjects were excluded if there were irreconcilable differences between the reported gender and the gender as determined from genotype analysis. A check was made for subject-identifier mismatch by means of expected genetic kinship within and between pedigrees and in some cases helped to resolve gender-mismatch issues.

For our heritability analysis, there were two sets of subjects genotyped on different chips. One set was comprised of the RSTR, LTBI, and active TB subjects genotyped on the MEGA^EX^ chip described above. However, in order to include the phenotypes we needed to examine the full range of our TB comparisons representing different transitions in pathogenesis, we had to combine the genotype data from the aforementioned subjects with a set of subjects that used a different genotyping chip into a single large genetic relatedness matrix (GRM) for use in our heritability estimates. These set of subjects included 201 additional active TB cases, 15 RSTR subjects, 32 LTBI subjects, and 233 “No Active TB” subjects. This second genotyping chip was the Illumina HumanOmni5 microarray, which comprises ∼4.3 million genome wide markers, and offers high coverage of common genetic variation even for African populations [32]. Genotype calling and quality control were performed as previously described [32]. After QC and filtering by minor allele frequency (MAF > 0.05), genotyping call rate of 98%, and Hardy-Weinberg equilibrium threshold of *p* < 10^−6^ , a total of 337, 566 SNPs passed QC and overlapped between the two genotyping chips; QC criteria differed slightly because of the difference in genotyping chips. These 337, 566 SNPs were used for the heritability analyses.

### 2.3 Kinship and PC Generation

Kinship and principal component (PC) generation was performed separately for the two analyses. For the GRM-based heritability analysis, we computed principal components but did not include them based on: 1.) prior studies using the same genotype data showed that there is not substantial population sub-structure or batch effects between the two chips [33]; and 2.) the plots of PC1 vs. PC2 that showed a lack of clustering in our combined genotype data used in the heritability estimation (Supplemental Figure 1) previous literature showing that it is not necessary to include kinship estimation in a genetic related matrix based heritability estimation [34]. Nonetheless, we performed sensitivity analyses to show that the inclusion of these PC's did not change our final estimates (Supplemental Table 1). In the GWAS analysis described above, to correct for relatedness among individuals in our sample due to population and family structure, we carried out a principal components analysis (PCA) and estimated kinship among the 263 RSTR or LTBI individuals passing QC during the association analysis (see below). A genome-wide panel of 135,859 common (MAF ≥ 0.05) independent (pairwise *r*^2^ < 0.1) variants passing marker QC from the MEGA^EX^ panel was chosen for the PC and kinship generation. We calculated PCs using PC-AiR and kinship using PC-Relate in the R, Genesis package [35]. The Genesis package pipeline for generating PCs and kinship first determines relatedness via an initial KING estimated kinship matrix. It then determines the population structure from the unrelated subset of the sample (determined by a KING-estimated kinship threshold of 0.0221) and projects the loadings onto the subset of related subjects to obtain PCs that account for population structure with family structure removed. Genesis then re-calculates kinship using PC-Relate by adjusting for the PCs generated from PC-AiR to get an estimate of family structure with population structure removed. The total number of PCs used in the GWAS was determined by examining the elbow plot of PCs and selecting the count where the variation explained by additional PCs was minimal (Supplemental Figure 1).

### 2.4 Statistics

For our GWAS we employed a score-based association test in a mixed model framework that allows for the inclusion of a polygenic random effect (e.g. a genetic relationship matrix). We used the assocTestSingle function in the Genesis package to conduct the genome-wide association analysis. Because our phenotype is binary (RSTR/LTBI) Genesis uses the penalized quasi-likelihood (PQL) approximation to the generalized linear mixed model (GLMM) to fit the specified model, following the procedure of GMMAT [36]. We controlled for population structure and family structure in our model including the first two principal components (see above) as fixed effects and the genetic relationship matrix (GRM, see above) as a random effect, respectively. Two PCs were selected based on an elbow plot of the variation explained as a function of the number of clusters in our data. Additional covariates included in the model were age and sex. In order to interrogate the role of variants that did not meet the traditional GWAS p-value threshold but could nonetheless be truly associated, we used the p=5 × 10^−5^ threshold as “suggestive” of significance thereby allowing us to examine variants that may have important regulatory or biological function, despite not reaching the typical GWAS threshold of significance (i.e. p=5 × 10^−8^) [37]. In previous studies, SNPs below the GWAS threshold in one study have successfully replicated when tested again using the same phenotype in subsequent studies, revealing additional variants that are associated with the phenotype of interest. Specifically, SNPs that meet the suggestive threshold but not the GWAS threshold in some cases have at times been shown to be GWAS significant (i.e. p<5 × 10^−8^) after further study with a larger sample size [38,39]. Thus, it may be useful and informative to examine the biological role of SNPs that are in this range of p values, especially with regard to a potential regulatory role and keeping in mind that we had a relatively small sample size [37].

For the heritability analyses, a genetic relatedness matrix was constructed for restricted maximum likelihood estimation (REML) [40] in GCTA software to estimate the genome-wide heritability of three different phenotypes: 1.) susceptibility to active tuberculosis (compared to latent TB and uninfected); 2.) RSTR (compared to LTBI); and 3.) of progression to active TB (compared to latent TB). GCTA estimates the variance explained by all the SNPs across the whole genome for a complex trait [40,41]; all references to heritability estimates derived for this paper used this methodology and are simply referred to as “heritability” hereafter.

In order to account for the impact of age, sex, and HIV status on these heritability estimates we first ran all heritability estimates unadjusted. We then computed heritability estimates that were adjusted for age, sex, and HIV status as covariates. Further, as it is possible that the inclusion of HIV+ subjects might affect the heritability estimates in ways that are not accurately reflected by the inclusion of the three aforementioned covariates, we ran a stratified analysis that included only HIV- subjects. This allows us to observe how these covariates affect our estimates and to compute estimates that are less affected by bias.

### 2.5 Functional annotation

We prioritized loci based on a scoring scheme that combines various forms of evidence regarding putative functionality. This was done in order to provide semi-quantitative evidence that may indicate a potential functional role for our SNPs that did meet the GWAS threshold but showed low enough p-values to warrant further interrogation [42]. The scheme assigned 1 point to each locus for each of the following categories: having >1 SNP below the suggestive threshold at the locus with an extra point for loci with SNPs having a p-value < 5 × 10^−6^; evidence for a regulatory role as shown by RegulomeDB; serving as an eQTL based on FUMA; and biological relevance to TB based on prior literature. Because the majority of our findings are intergenic and/or fall in noncoding regions, we relied on the annotation tool FUMA version 1.36 for mapping our variants to genes based on genomic proximity, eQTL evidence and chromatin interaction evidence [43]. Default settings in FUMA were used, except for tissue specificity. FUMA does not distinguish between cis and trans-eQTL's in their annotations. However, we considered any locus within 1 Mb of the gene being regulated (and on the same chromosome) as a cis-eQTL, a definition based on prior literature surrounding eQTL function [44,45]. We hypothesized that gene expression and regulation would be most relevant in lung, immune cells and blood and thus, focused on eQTL and chromatin interaction evidence in these target tissues. FUMA and RegulomeDB v2 were used for eQTL identification and to further examine chromatin state evidence and specify enhancer or transcription evidence within lung, immune cells, and blood. GeneCards was used to elucidate gene function and evidence from the literature was used to elucidate a potential biological role for genes in the context of resistance to TB [46].

To further enrich our results and yield greater biological insight, we utilized FUMA GWAS’ GENE2FUNC feature for the genes represented in our GWAS summary statistics. This feature maps GWAS summary statistics to genes, and then provides gene set or pathway enrichments based on gene sets from MsigDB, KEGG, WikiPathways, and the GWAS Catalog. This function tests our mapped genes for enrichment using pre-established databases of gene sets from prior gene set enrichment analysis (GSEA) analyses using hypergeometric mean pathway analysis and adjusts the p-values for significance based on the Benjamini-Hochberg method (i.e. FDR) using the number of data sources of tested gene sets. FUMA reports gene sets with adjusted P-value ≤ 0.05 and the number of genes that overlap with the gene set > 1 [43].

Additionally, STRING network analyses were used to assess if there were protein-protein interactions between the downstream products of the genes identified in our analyses. This allows us to look for common networks or interactions that provide further biological insight into our results. STRING can determine if there is a greater degree of relatedness than expected among our results and use this to determine overall protein-protein interaction enrichment as well as enrichment for specific networks, gene ontologies, and previously published works [47].

### 2.6 Sample size estimation

There was no power calculation or sample size calculation done prior to the onset of this study. The sample was determined by which study participants had sufficient DNA and clinical data available for the planned analyses, using the entire sample from the follow-up study [31].

### 2.7 Ethics

The study was approved by the National AIDS Research Committee, The Uganda National Council on Science and Technology (IRB number ARC 014), and the institutional review board at University Hospitals Cleveland Medical Center (IRB number 10-01-25). Written informed consent was obtained from all individuals in the study. The Ugandan IRB has restricted availability of these data; investigators interested in obtaining these data must contact Dr. Sudha Iyengar, ski@case.edu, chair of the Data Access Committee for this study.

### 2.8 Role of the Funding source

The funding source had no role in the study design, statistical analysis, or interpretation of data.

## 3 Results

### 3.1 Study Population

We examined 908 people in the final combined sample (Table 1), including 263 RSTR and LTBI individuals (74 RSTR and 189 LTBI) with high quality genotyping data and follow-up clinical information (and thus used in the GWAS analysis), who were all HIV-negative and were similar with respect to age and sex (Table 2) [31]. 350 active TB cases and 233 “No active TB” subjects were in the heritability analysis, but not included in the association (GWAS) analysis. The RSTR and LTBI categories for our heritability estimation included a larger number of subjects (97 and 228, respectively) than the GWAS analysis. The differences are due to the inclusion of HIV+ subjects and subjects who were genotyped using a different chip (i.e. the Omni5 chip) in the heritability analysis.

**Table 1:** Cohort Characteristics for Heritability

	**RSTR N=97**	**LTBI N=228**	**Active TB N=350**	**No Active TB N=233**
**Age**	23.6 (11.0)	24.4 (9.7)	27.5 (10.3)	16.2 (13.7)
**Females**	45 (46.4%)	88 (49.2%)	192 (54.9%)	123 (52.7%)
**Males**	52 (53.6%)	91 (50.8%)	158 (45.1%)	110 (47.2%)
**HIV+**	13 (13.4%)	6 (3.4%)	61 (17.5%)	15 (6.5%)
**HIV-**	84 (86.6%)	173 (96.6%)	288 (82.5%)	214 (91.8%)

"No active TB" refers to subjects who did not have long-term follow up data including IGRA. This includes subjects who were TST+ or TST- during the original study, but could not be categorized without IGRA and long-term data.

**Table 2:** Cohort Characteristics for GWAS

Variable	Total	RSTR	LTBI	p*
Sample Size	263	74	189	-
Female	125 (47.5%)	38 (51.4%)	87 (46.0%)	0.52
Age, years	23.58 ± 8.9	22.5 ± 8.8	24.0 ± 8.9	0.06
Age range, years	2-55	14-66	14-66	-

Values are shown as N (%) or as mean ± SD.

⁎ Comparisons between RSTR and LTBI were made using Pearson's χ^2^ test and for continuous variables using the non-parametric Wilcoxon rank sum test.

### 3.2 Heritability Analysis

For our analysis of active TB relative to all other phenotypes (i.e. LTBI, RSTR and no infection), the heritability (i.e. the percentage of variation in the outcome explained by all variants present on the GWAS chip) without adjustment was 28.7% (Table 3). The heritability was 50.9% for RSTR vs. LTBI, 45.2% for RSTR vs. LTBI and active TB, and 56.0% for active TB vs. LTBI. Adjusting for age, sex, and HIV status increased the estimated heritability to 37.1% for active TB relative to LTBI or “no active TB” (compared to 28.7% in unadjusted analysis) and lowered the heritability for RSTR vs. LTBI slightly to 48.3% (compared to 50.9% in unadjusted analysis). The adjustments had no effect on the heritability for RSTR vs. LTBI and active TB (45.2% unadjusted vs. 45.2% adjusted), and of active TB vs. LTBI (56.4% adjusted vs 56.0% unadjusted). This analysis shows that stricter phenotypic comparisons yielded higher heritability estimates, demonstrating the importance of clear phenotype definition.

**Table 3:** Heritability Estimates.

**Phenotype Definition**	**Unadjusted**	**Adjusted**⁑	**HIV-Only**ǂ
Active TB vs. LTBI and “no active TB”*	28.7%	37.1%	38.7%
**TB vs. LTBI**	56.0%	56.4%	55.2%
**RSTR vs. LTBI**	50.9%	48.3%	50.4%
**RSTR vs. LTBI and Active TB**	45.2%	45.2%	45.8%

⁎ “no active TB” refers to both RSTRs and subjects who did not have long-term follow up data including IGRA. This includes subjects who were TST+ or TST- during the original study, but could not be categorized without IGRA and long-term data and are referred to as “no active TB” earlier in the document.

⁑ “adjusted” refers to estimates that were adjusted for age, sex, and HIV status. For the RSTR vs. LTBI, the age at which they were distinguished as RSTR or LTBI in a sub-study was used. For the other estimates, the age at first presentation was used.

ǂ These estimates only included the HIV- subjects and were adjusted for age and sex

To examine whether the inclusion of HIV+ subjects had an effect on the estimates, we estimated heritability among the HIV- persons only. Our analysis stratified by HIV status (and adjusted for age and sex) showed that the heritability estimates were only slightly different when HIV+ subjects are removed but the effect was not large. For the HIV- subjects, the estimates were 38.7% for TB vs. all other phenotypes, 50.4% for RSTR vs. LTBI, 45.8% for RSTR vs. LTBI and active TB, and 56% for active TB vs. LTBI. Thus, our stratified analysis showed that the inclusion of HIV+ subjects in the heritability estimates did not change our outcome drastically compared to our adjusted estimates, implying that these estimates are not particularly sensitive to HIV status.

### 3.3 Association Analysis

**Figure 2:**
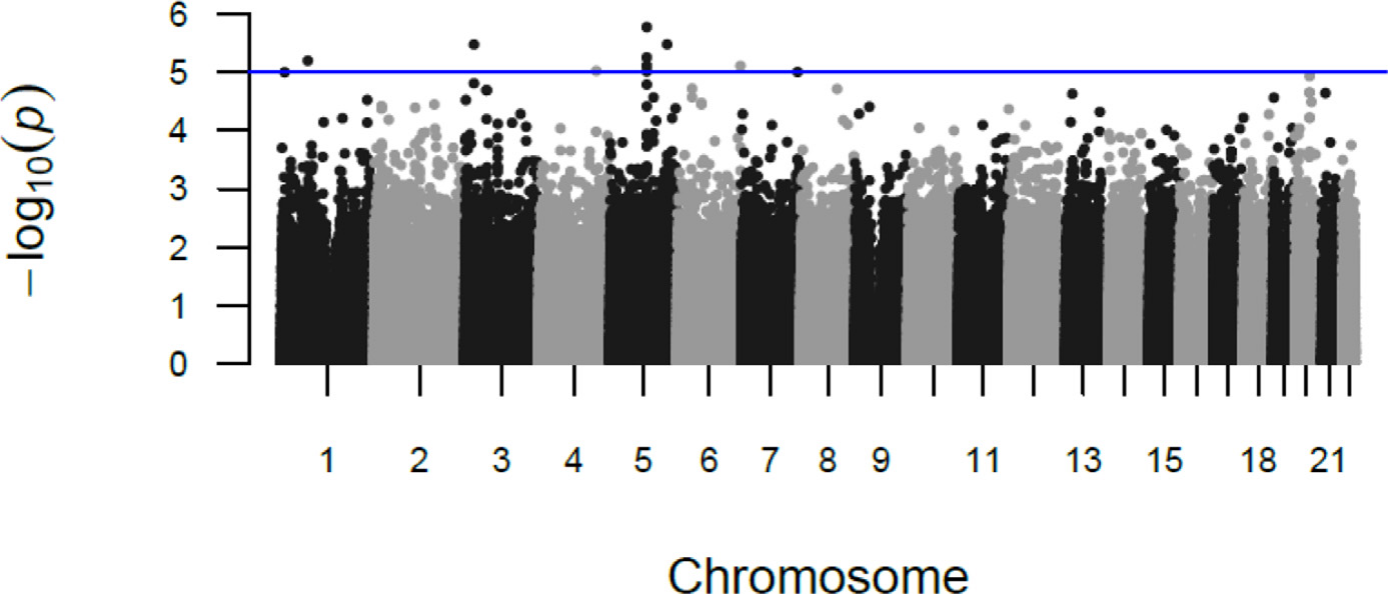
Manhattan Plot of P-Values for association between SNPs and RSTR phenotype (relative to LTBI) The GWAS utilized a score-based association test in a mixed model framework that incorporated a genetic relationship matrix. Model covariates included were age, sex and first two PCs. Sample size was 263, with 74 RSTRs and 189 LTBI subjects.

**Figure 3:**
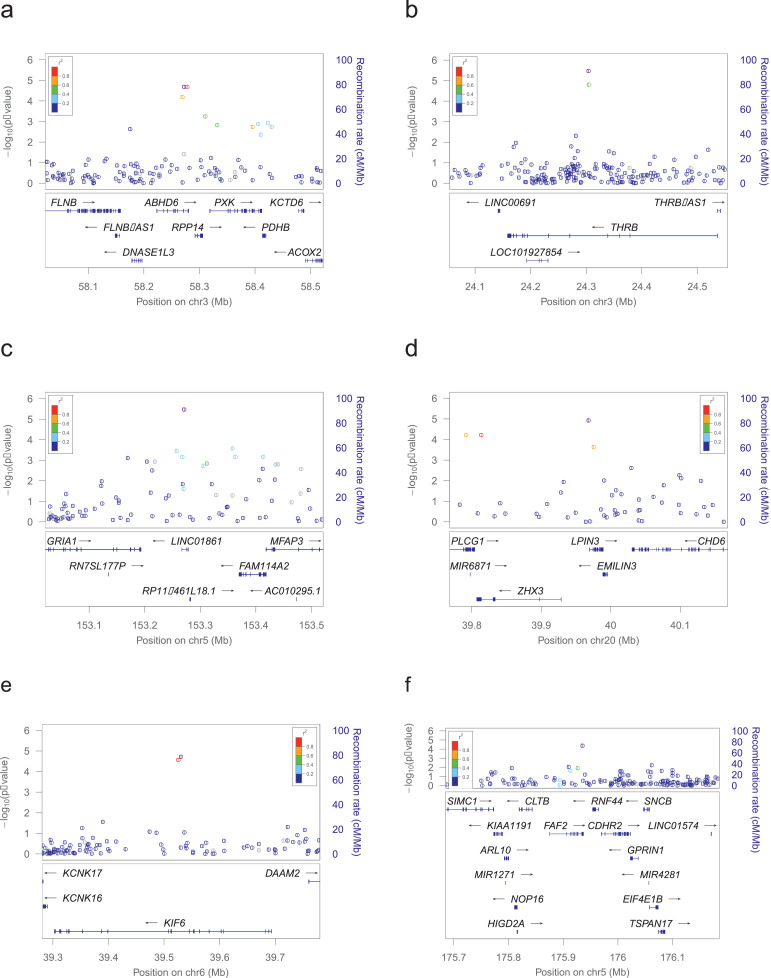
Locus Zoom plots for featured loci Locus Zoom plots for featured, significant loci. Region shown is the gene for which the top variant falls in, plus 200kb flanking. LD shown is for 1000 Genomes, November 2014, African population. a) ABHD region with top SNP rs9848072 (3:58272639). b) THRB region with top SNP rs78813564 (3:24304291). c) LINC01861 region with top SNP rs919222 (5:153271156). d) ZHX3/LPIN3 region with top SNP rs6072343 (20:39968188). e) KIF6 region with top SNP rs10484824 (6:39530428). f) FAF2 region with top SNP rs2963672 (5:175935316).

There were 40 SNPs spanning 27 loci with a suggestive association with RSTR vs LTBI ($p<5\times 10^{-5}$) (Figure 2, Supplemental Table 2). Supplemental Table 2 shows one line for each locus with at least one SNP showing a p-value below 5x10^-5^; the rsID and position is for the SNP with the lowest p-value within each locus. The plurality of associated SNPs were intronic (48%) or intergenic (38%). Sixty percent of these suggestive loci overlapped regions showing strong evidence for transcription and/or enhancer activity in lung, immune cell or blood tissues (as determined by RegulomeDB) and 30% harbored eQTLs in these relevant tissues (Supplemental Table 3). Using our scoring scheme for prioritizing SNPs, there was one locus with 4 points and five loci with 3 points (Supplemental Table 2). We have described these 6 loci (Table 4, Figure 3) in greater detail below.

**Table 4:** GWAS results for featured loci*.

Chr:Pos	Top SNP	Associated Gene	MAF	OR	95% CI for OR	Score Stat	Score Test P	Effect Allele
3:58272639	rs9848072	ABHD6	0.07	4.81	[2.34, 9.92]	4.26	2.06 × 10^−5^	G
3:24304291	rs78813564	THRB	0.057	8.12	[3.35, 19.51]	4.65	3.39 × 10^−6^	G
5:153271156	rs919222	LINC01861	0.388	0.385	[0.26, 0.58]	-4.65	3.38 × 10^−6^	A
20:39968188	rs6072343	ZHX3;LPIN3	0.0856	5.02	[2.43, 10.26]	4.38	1.19 × 10^−5^	A
6:39530428	rs10484824	KIF6	0.0875	4.57	[2.28, 9.18]	4.27	1.93 × 10^−5^	G
5:175935316	rs2963672	FAF2	0.19	3.06	[1.79, 5.23]	4.09	4.22 × 10^−5^	G

⁎ Featured loci all had P-values < 5 × 10^−5^ and were selected based on biological relevance to RSTR phenotype

The locus with the highest score in our scheme was a region on chromosome 3, including the *ABHD6* gene (Figure 3a). The top SNP in the region, rs9848072 (OR=4.8, score test p=2 × 10^−5^), falls in the last intron of *ABHD6* and the locus is a cis-eQTL for this gene. The region also harbours eQTLs for several other genes, *RPP14, ABHD6, PXK, DNASE1L, FLNB* and *PDHB*, in lung, immune cell and blood tissues. Analysing the 6 genes in a STRING network shows 2 edges and enrichment for endonuclease activity, actin binding, and hydrolase activity, acting on ester bonds (Supplemental Figure 2). The adjusted P-value for PPI enrichment, i.e. the statistical test showing that there are more protein-protein interactions than would be expected by chance alone, was p=4.2 × 10^−5^. It is possible that these genes are co-expressed as part of a gene network, which is why one SNP shows an association with all 6, despite mapping to only 1 of them (i.e. being a cis-eQTL for *ABHD6*).

The first locus with 3 points mapped to the *THRB* gene on chromosome 3, and encodes a thyroid hormone receptor [46] (Figure 3b). This locus showed strong evidence for regulation of transcription in the lung and contains an enhancer for *THRB* in lung tissue, according to RegulomeDB [48]. The second locus with 3 points was located on chromosome 5 and mapped to *LINC01861*, a long intergenic non-coding RNA gene (Figure 3c). This locus contains an cis-eQTL that is located within 0.1 Mb of and on the same chromosome as *MFAP3* and *FAM114A2*, two genes that previously showed evidence for co-expression in a micro-array analysis [49]. It is also an enhancer in immune cells [48]. The third locus with 3 points was located on chromosome 20 and is flanked by *ZHX3* and *LPIN3* (Figure 3d). It shows evidence of strong transcription in the lung and is a cis-eQTL for *CHD6*, a gene within 0.1 Mb on the same chromosome that encodes a DNA-dependent ATPase and is active in chromatin remodelling in response to oxidative stress [46]. The fourth locus was on chromosome 6 and maps to *KIF6* (Figure 3e). It also includes a cis-eQTL for *KIF6* that encodes a protein in the kinesins family. This family of proteins is responsible for intracellular transport of protein complexes [46].

A 5^th^ locus with 3 points was on chromosome 5 and mapped to *FAF2* (Figure 3f). The top SNP in the region, rs2963672, falls in the UTR3 within *FAF2* (OR=3.1, score test p=4 × 10^−5^) and is a cis-eQTL for *FAF2*. The region also harbours eQTLs for several other genes, *ARL10, NOP16, HIGD2A, RNF44, CDHR2* and *GPRIN1* in blood(48). This region shows strong evidence for regulation of transcription of *FAF2* in lung, blood and immune tissue in RegulomeDB. A STRING network analysis showed a number of edges and similar to our top SNP, this may represent a network of genes with associated expression (PPI enrichment p=2.6 × 10^−7^) [47] (Supplemental Figure 3).

We also examined regions that have been previously associated with TST reactivity or LTBI in previous genome-wide and candidate gene studies (Supplemental Table 4). While we were unable to strictly replicate association within specific candidate genes because we did not type the same SNPs, we did observe association with the RSTR phenotype in chromosomes 2 and 5 originally identified by a genome-wide linkage study in Ugandans (score test p=1 × 10^−4^, both regions), the TST2 locus originally linked to TST reactivity (score test p=1.66 × 10^−4^), and the *IL9* region associated with TST reactivity in an HIV+ cohort (score test p=6.99 × 10^−4^).

**Figure 4:**
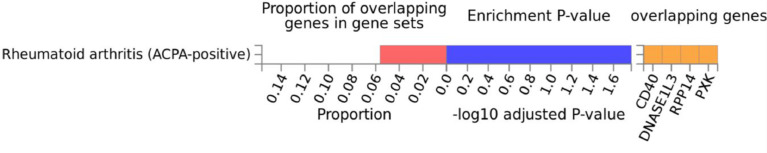
Gene Set Enrichment for GWAS Summary Statistics Gene set enrichment done using FUMA GWAS’ GENE2FUNC feature for the mapped genes represented in our GWAS summary statistics. Gene sets considered were from MsigDB, KEGG, WikiPathways, and the GWAS Catalog. Depicted is the significant result (FDR p<0.05) from the GWAS Catalog.

In our gene enrichment analysis of SNPs that were P<1x10^-5^ for association, our SNPs showed enrichment for only one biological pathway, the anti-citrullinated protein antibody positive rheumatoid arthritis (ACPA positive RA) pathway from the GWAS catalog, with a p-value of 9.36 × 10^−6^ **(**FDR-adjusted p=1.70 × 10^−2^) as determined by GENE2FUNC. This was based on four genes that showed association with the RSTR phenotype and are also found in the ACPA positive RA pathway: *CD40, DNASE1L3, RPP14, PXK* (Figure 4).

## 4 Discussion

Overall, our results show that the RSTR phenotype has a higher heritability than previously reported and that specific variants associated with RSTR likely serve regulatory roles in lung and immune cells. We posit that the higher heritability estimate seen in this study compared to previous studies is due to a purer phenotypic definition, with less misclassification. Further, we were able to confirm exposure to MTB infection, greatly reducing false negatives in our data. We previously found that the prevalence of the RSTR phenotype varies as a function of follow-up time and use of TST as compared to IGRA [6,8,9,50]. Importantly, studies without a long duration of follow-up will likely misclassify subjects due to subjects converting to TST positivity after the follow-up has concluded. In addition, subjects can revert from positive to negative with TST/IGRA tests which can lead to misclassification. As the RSTRs included in this study had an average of 9 years follow-up after initial TB exposure and both TST and IGRA were utilized in the clinical definition we were able to assign the RSTR status with little or no ambiguity. This is the first genetic analysis to utilize this strict definition of resistance to infection. The importance of strict definition for heritability estimation was also seen in our TB heritability estimates. When including subjects without long-term follow-up and/or without confirmed RSTR status, the heritability estimate was lower. Our strict TB vs. LTBI contrast yielded a higher heritability estimate, likely reflecting strong genetic influence on progression; because MTB exposure is documented, the influence of misclassification by lack of exposure on heritability can be minimized.

While our GWAS results did not identify any single variant that is significant at the traditional “GWAS threshold,” our results are consistent with a number of putatively associating variants with regulatory roles consistent with the RSTR phenotype. The SNPs that were significant at a p<1 × 10^−5^ level are mostly in regions that have evidence of a regulatory function in RegulomeDB. Many of these SNPs were eQTLs and some exhibit biological relevance as judged by tissue specific effects.

*ABHD6*, our top locus using the scoring scheme we developed, produces a lipase that can degrade bis monoacylglycerol phosphate (BMP) and constitutes the major enzyme for BMP catabolism. BMP is expressed in the late endosomes and lysosomes of phagocytosing macrophages [12]. In most mammalian cells, BMP levels are low, comprising only about 1–2% of total phospholipids. However, BMP constitutes 16% of the total phospholipids in lung alveolar macrophages [13]. Additionally, the gene *DNASE1L3*, a gene for which the *ABHD6* SNP is a cis-eQTL, codes for a protein that plays a key role in degrading neutrophil extracellular traps (NETs). NETs are mainly composed of DNA fibres and are released by neutrophils to bind pathogens during inflammation. NETs may play a key role in the pathway responsible for non-specific inflammation and tissue destruction in pulmonary TB [14]. Therefore, the SNP in *ABHD6* is potentially relevant to TB development through two distinct biological processes.

*FAF2*, one of our second highest scoring loci, is part of the innate immune system and regulates endoplasmic reticulum-associated degradation (ERAD), a system for ubiquitin-dependent degradation of misfolded proteins [46]. FAF2 controls the steady-state expression of the IGF1R receptor, thus indirectly regulates the insulin-like growth factor receptor signalling pathway [51]. IGF-I has been shown to contribute to the maintenance of *Mycobacterium leprae* persistence in the host, reinforcing a key role for IGF-I in leprosy pathogenesis [52]. It has been found that blocking IGF-I signalling rescues antimicrobial activity in *M. leprae* (ML)-infected macrophages and furthermore, knockdown of IGF-1R rescues antimicrobial activity in ML-infected human macrophages [23]. These cytokines and processes are similar between the *M. leprae* response and the response to MTB infection and adds plausibility that these genes could play important roles in the RSTR phenotype.

Four of our loci mapped to genes that show enrichment for the rheumatoid arthritis (RA) pathway based on the GWAS catalogue. The component genes of the RA pathway as well as the pathway itself are known to be important in susceptibility to infectious disease. RA is an inflammatory disease characterized by increased levels of pro-inflammatory cytokines (particularly TNF-α) that are important to host immune response in TB [53]. Many RA drugs are anti-TNF biologics, and depression of normally important cytokines among RA patients receiving treatment leads to a susceptibility to developing active TB or a re-activation among LTBI patients [54]. Previous immunological studies have shown the importance of TNF (and the other Th1 cytokines) in the TB response [55]. A previous meta-analysis of hundreds of TB susceptibility studies showed enrichment for the RA pathway when the meta-analytic summary statistics were analysed for pathway enrichment [24]. Further, the RA pathway has been shown to be an important part of the alveolar inflammation response to infection; alveolar macrophages are the first target of infection for MTB, and they have previously been discussed as a possible mediator of the RSTR phenotype [56,57]. Thus, the results presented in this study in conjunction with previous literature are consistent with genetic variants in the pro-inflammatory Th1 cytokine response, which are also grouped together in the RA pathway, being associated with the RSTR phenotype and resistance to MTB infection.

This study was not without limitations. We were not able to detect any variants that were associated with the RSTR phenotype at the GWAS threshold of p<5 × 10^−8^ but this may be due to small sample size and the inability to replicate findings due to the lack of an additional cohort with a similarly strict phenotype definition. Further, while family-based association testing may have a lower power to detect an association, we were able to control for exposure between RSTR and LTBI individuals whereas typical case-control studies do not. Despite these limitations, our results identified eQTLs, enhancers, and other regulatory functions that are biologically plausible in the context of resistance to infection by MTB. Additionally, we were able to utilize a larger sample size for our heritability estimates and we were able to demonstrate that the RSTR phenotype has a high heritability despite a lack of individually associating SNPs at the GWAS level. This may imply that the RSTR phenotype is influenced by a variety of genes and variants rather than individual SNPs or genes. Lastly, while the proportion of HIV-infected individuals in this sample is small, it is possible that low CD4 counts could affect susceptibility to TB disease or acquisition of MTB infection. Unfortunately, most of these HIV-infected individuals were enrolled in the study prior to the availability of anti-retrovirals in Uganda, so CD4 count and viral load assays were not routinely conducted, so we are unable to assess the impact of these relevant covariates on our outcomes in this cohort.

Overall, these findings demonstrate how a purer clinical phenotype can yield higher, and probably more accurate, heritability estimates and identify potential new candidate genes for resistance to MTB infection. Our observation that many of these associated SNPs have regulatory functions add to our existing understanding of how genetic variants, including those from previous studies of the RSTR phenotype, influence resistance to MTB infection and build on our previous knowledge of TB pathogenesis. Genetic studies like this provide additional insight into alternate immune responses seen between RSTRs and LTBI [7].

## Contributors

C.M.S, M.L.M, and T.R.H conceived of the design and analysis. W.H.B, H.M-K, M.N. and C.M.S. oversaw the subject recruitment and characterization. M.L.M. and P.B. conducted the statistical analysis, L.M. oversaw the data collection and constructed clinical datasets, and C.M.S. and S.M.W. supervised the statistical analyses. M.L.M., P.B., and C.M.S. verified the underlying data. M.L.M. and P.B. drafted the initial version of the manuscript, and all authors had full access to all of the data in this paper and edited and approved the final version of the manuscript.

## Funding

This work was funded by grants from the Bill and Melinda Gates Foundation grant OPP1151836 (to T.R.H., W.H.B, C.M.S., H.M.K.); R01AI124348 (to W.H.B, T.R.H., C.M.S., H.M.K.), U01AI115642 (to W.H.B, T.R.H., C.M.S., H.M.K), 4R33AI138272 (WHB, TRH, SMC, HMK), and Tuberculosis Research Unit (grant N01-AI95383 and HHSN266200700022C/ N01-AI70022, to W.H.B, C.M.S, H.M.K, and T.R.H.). MLM was supported by grants T32 HL007567, T32 GM007250, and TL1 TR002549.

## Data Sharing Statement

Because of restrictions on the study placed by the Ugandan ethical review boards, the GWAS data are not available for broad sharing. Investigators who are interested in obtaining the data must apply to the Data Access Committee, chaired by Dr. Sudha Iyengar (ski@case.edu) with a data analysis plan, data security plan, and plan to protect human subjects.

## Declaration of Competing Interest

The authors have no conflicts of interest to report.

## Appendix Supplementary materials

**Supplemental Table 1.** Heritability analysis including PCs as covariates Image, application 1

**Supplemental Table 2.** Loci and lead SNPs with P<5e-05 for Association with RSTR Phenotype Image, application 2

**Supplemental Table 3**. eQTL's Located in Loci with P<5e-05 for Association with RSTR Phenotype Image, application 3

**Supplemental Table 4.** Loci Previously Associated with RSTR Phenotype and Accompanying Summary Statistics in Current Study Image, application 4
