# Supplementary material for: Resistance to TST/IGRA conversion in Uganda: Heritability and Genome-Wide Association Study
Source: eBioMedicine. 2021 Dec 4;74:103727. doi: 10.1016/j.ebiom.2021.103727 (PMC8652006; doi:10.1016/j.ebiom.2021.103727)

**Supplemental Figure 1. Principal Component Plots**

|  |  |
| --- | --- |
|  |   **Elbow plot for selecting PCs** |

**Supplemental Figure 2. STRING diagram for eQTLs**


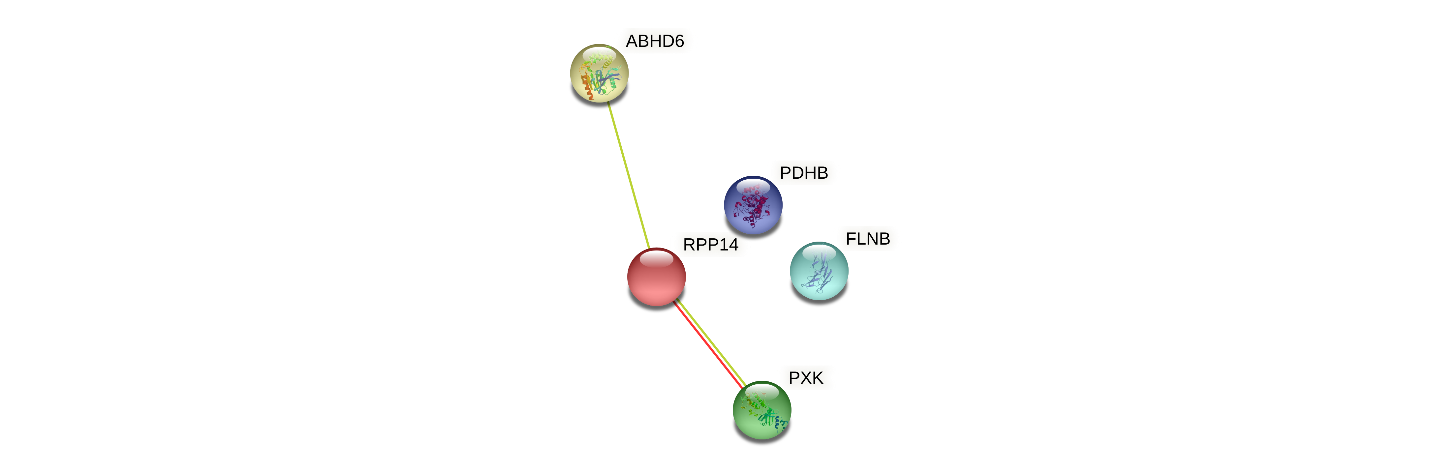


**Supplemental Figure 3. STRING diagram for eQTLs**


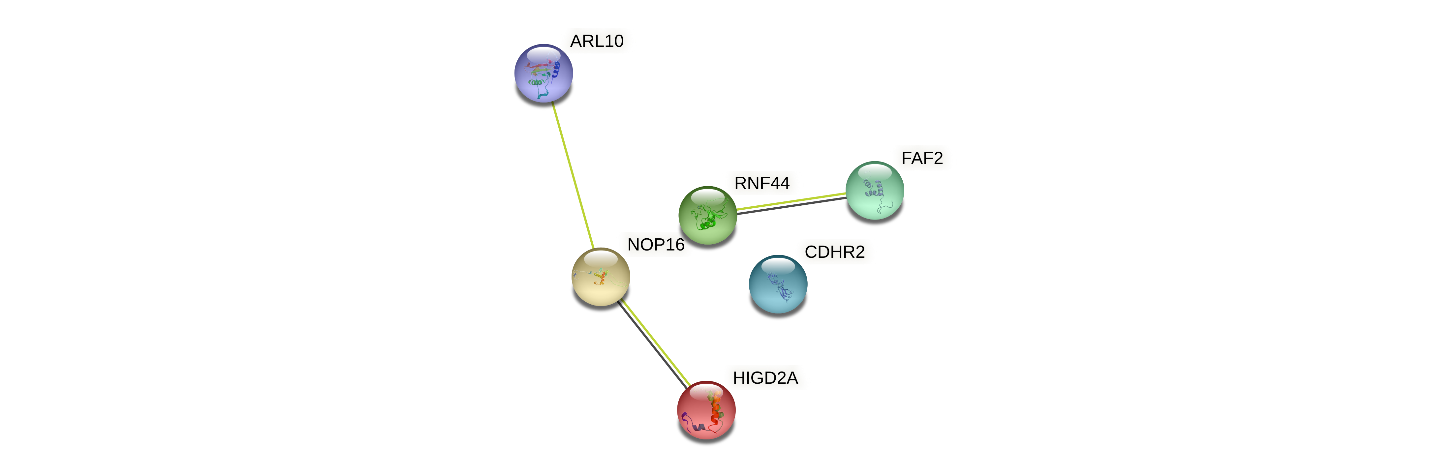

Supplement: Supplementary file 1 — Supplemental Table 1. Heritability analysis including PCs as covariates [file mmc1.docx]
