# Supplementary material for: Resistance to TST/IGRA conversion in Uganda: Heritability and Genome-Wide Association Study
Source: eBioMedicine. 2021 Dec 4;74:103727. doi: 10.1016/j.ebiom.2021.103727 (PMC8652006; doi:10.1016/j.ebiom.2021.103727)
Supplement: Supplementary file 2 — Supplemental Table 2. Loci and lead SNPs with P<5e-05 for Association with RSTR Phenotype [file mmc2.docx]

**Supplemental Table 1. Heritability estimates adjusted for PCs and other covariates**

| **Phenotype Definition** | **With PC's (HIV- only, adjusted for age and sex)** |
| --- | --- |
| **Active TB vs. LTBI and "no active TB"** | 40.9% |
| **RSTR vs. LTBI** | 51.8% |
| **RSTR vs. LTBI and Active TB** | 46.7% |
| **TB vs. LTBI** | 57.0% |
