# Supplementary material for: Resistance to TST/IGRA conversion in Uganda: Heritability and Genome-Wide Association Study
Source: eBioMedicine. 2021 Dec 4;74:103727. doi: 10.1016/j.ebiom.2021.103727 (PMC8652006; doi:10.1016/j.ebiom.2021.103727)
Supplement: Supplementary file 3 — Supplemental Table 3. eQTL's Located in Loci with P<5e-05 for Association with RSTR Phenotype [file mmc3.pdf]

| Lead SNP | CHR:POS | #SNPs | >1 snp pts | relevance points | Genes/FlankingGenes | MAF | Score_pval | p-value points | eQTL Genes | eQTL-points | RegulomeDB regulatory evidence | Regulome points | score |
|---|---|---|---|---|---|---|---|---|---|---|---|---|---|
| rs9848072 | 3:58272639 | 2 | 1 | 1 | ABHD6 | 0.07 | 2.06E-05 | 0 | PDHB, PXK, RPP14, ABHD6, DNASE1L3, FLNB | 1 | strong transcription in lung, blood and immune cells. | 1 | 4 |
| rs78813564 | 3:24304291 | 2 | 1 | 0 | THRB | 0.057 | 3.39E-06 | 1 | | 0 | strong transcription in lung; enhancer in blood. | 1 | 3 |
| rs919222 | 5:153271156 | 1 | 0 | 0 | LINC01861 | 0.388 | 3.38E-06 | 1 | FAM114A2, MFAP3 | 1 | enhancer in immune cells. | 1 | 3 |
| rs6072343 | 20:39968188 | 3 | 1 | 0 | ZHX3; LPIN3 | 0.086 | 1.19E-05 | 0 | CHD6 | 1 | strong transcription in lung. | 1 | 3 |
| rs10484824 | 6:39530428 | 2 | 1 | 0 | KIF6 | 0.087 | 1.93E-05 | 0 | KIF6 | 1 | strong transcription in lung. | 1 | 3 |
| rs2963672 | 5:175935316 | 1 | 0 | 1 | FAF2 | 0.188 | 4.22E-05 | 0 | ARL10, NOP16, HIGD2A, FAF2,RNF44, CDHR2,GPRIN1 | 1 | strong transcription in lung, blood, immune cells. | 1 | 3 |
| rs2461966 | 5:98733594 | 6 | 1 | 0 | LOC100289230; LINC02113 | 0.18 | 1.71E-06 | 1 | | 0 | | 0 | 2 |
| rs12614401 | 2:19961899 | 3 | 1 | 0 | OSR1; LINC00954 | 0.163 | 3.83E-05 | 0 | | 0 | strong transcription in lung. | 1 | 2 |
| rs1482463 | 6:64589465 | 2 | 1 | 0 | EYS | 0.473 | 3.39E-05 | 0 | LGSN | 1 | | 0 | 2 |
| rs17369958 | 4:154459958 | 1 | 0 | 1 | TMEM131L | 0.067 | 9.59E-06 | 0 | | 0 | Active TSS in lung; strong transcription in immune; enhancer in blood. | 1 | 2 |
| rs10480299 | 7:151405818 | 1 | 0 | 1 | PRKAG2 | 0.297 | 1.00E-05 | 0 | | 0 | strong transcription in lung, blood, immune cells. | 1 | 2 |
| rs2275457 | 1:9427869 | 1 | 0 | 0 | SPSB1 | 0.394 | 1.02E-05 | 0 | | 1 | strong transcription in lung, blood and immune | 1 | 2 |
| rs1321001 | 20:44816736 | 1 | 0 | 1 | CDH22 | 0.443 | 3.26E-05 | 0 | CD40 | 1 | | 0 | 2 |
| rs10774291 | 12:634632 | 1 | 0 | 0 | B4GALNT3 | 0.454 | 4.34E-05 | 0 | B4GALNT3, NINJ2 | 1 | enhancer in immune cells | 1 | 2 |
| rs10075994 | 5:116750609 | 1 | 0 | 0 | LINC00992 | 0.093 | 2.74E-05 | 0 | | 0 | strong transcription in lung. | 1 | 1 |
| rs3829669 | 19:3371320 | 1 | 0 | 0 | NFIC | 0.125 | 2.77E-05 | 0 | | 0 | Active TSS in immune cells. Strong transcription in blood and lung. | 1 | 1 |
| rs853005 | 1:230651554 | 1 | 0 | 0 | PGBD5; LINC01737 | 0.055 | 3.02E-05 | 0 | | 0 | strong transcription in lung and blood | 1 | 1 |
| rs113880609 | 2:109915151 | 1 | 0 | 0 | SH3RF3 | 0.067 | 4.11E-05 | 0 | | 0 | strong transcription in lung. | 1 | 1 |
| rs76143195 | 13:110913564 | 1 | 0 | 0 | COL4A1 | 0.207 | 4.83E-05 | 0 | | 0 | strong transcription in lung | 1 | 1 |
| rs11209714 | 1:71371697 | 1 | 0 | 0 | PTGER3 | 0.373 | 6.42E-06 | 0 | | 0 | | 0 | 0 |
| rs79743909 | 6:169190085 | 1 | 0 | 0 | SMOC2; LOC105378146 | 0.046 | 7.89E-06 | 0 | | 0 | | 0 | 0 |
| rs1835742 | 8:98187086 | 1 | 0 | 0 | LOC101927066 | 0.241 | 1.96E-05 | 0 | | 0 | | 0 | 0 |
| rs258645 | 21:19396213 | 1 | 0 | 0 | CHODL | 0.078 | 2.31E-05 | 0 | | 0 | | 0 | 0 |
| rs74045367 | 13:37148580 | 1 | 0 | 0 | CCNA1; SERTM1 | 0.108 | 2.38E-05 | 0 | | 0 | | 0 | 0 |
| rs2619559 | 3:2647457 | 1 | 0 | 0 | CNTN4 | 0.327 | 3.03E-05 | 0 | | 0 | | 0 | 0 |
| rs113151125 | 2:161369243 | 1 | 0 | 0 | RBMS1; TANK | 0.078 | 3.61E-05 | 0 | | 0 | | 0 | 0 |
| rs570941 | 9:38273626 | 1 | 0 | 0 | SHB; ALDH1B1 | 0.16 | 3.96E-05 | 0 | | 0 | | 0 | 0 |
