# Supplementary material for: Resistance to TST/IGRA conversion in Uganda: Heritability and Genome-Wide Association Study
Source: eBioMedicine. 2021 Dec 4;74:103727. doi: 10.1016/j.ebiom.2021.103727 (PMC8652006; doi:10.1016/j.ebiom.2021.103727)
Supplement: Supplementary file 4 — Supplemental Table 4. Loci Previously Associated with RSTR Phenotype and Accompanying Summary Statistics in Current Study [file mmc4.pdf]

**Type of study**	**Gene/Locus**	**Phenotype**	**Population(s)**	**References**	**SNPs**	**Result**	**# SNPs with P<0.05**
Candidate gene	*ULK1*	TST positivity	Seattle	(Horne, Graustein et al. 2016, Shah, Musvosvi et al. 2017)	rs12297124 and rs7300908	Untested	
	SLC6A3	RSTR	Uganda	(Hall, Igo et al. 2015)	rs409588, rs456082, rs464061	p=0.9, Untested, Untested	
	IL10	TST positivity	Ghana	(Thye, Browne et al. 2009)	rs6703630, rs1800896, rs1800871	Untested, 0.2, 0.31	
Genome-wide	2q21-q24 (*GTDC1* and *ZEB2*)	RSTR (longitudinal)	Uganda	(Stein, Zalwango et al. 2008) (Cobat, Gallant et al. 2009)	rs74533435	1.09E-04	404
	5p13-q22			(Igo, Hall et al. 2018)	rs62375908	1.12E-04	885
	11p14 (*TST1*)	TST positivity (cross-sectional)	South Africa	(Cobat, Gallant et al. 2009)	rs293983	1.20E-03	67
	5p15 (*TST2*)	Reactivity (quantitative)		(Cobat, Hoal et al. 2013)	rs56345976	1.66E-04	346
	5q31 (including *IL9*)	TST positivity and reactivity	Tanzania and Uganda	(Sobota, Stein et al. 2017)	rs466157	6.99E-04	155
	10q26.2	TST and IGRA positivity	South Africa, France, Vietnam	(Quistrebet et al. pre-print)	rs17155120	6.20E-01	
